# On the Effect of Planetary Stable Isotope Compositions on Growth and Survival of Terrestrial Organisms

**DOI:** 10.1371/journal.pone.0169296

**Published:** 2017-01-04

**Authors:** Xueshu Xie, Roman A. Zubarev

**Affiliations:** Division of Physiological Chemistry I, Department of Medical Biochemistry and Biophysics, Karolinska Institutet, Stockholm, Sweden; National Institutes of Health, UNITED STATES

## Abstract

Isotopic compositions of reactants affect the rates of chemical and biochemical reactions. Usually it is assumed that heavy stable isotope enrichment leads to progressively slower reactions. Yet the effect of stable isotopes may be nonlinear, as exemplified by the “isotopic resonance” phenomenon. Since the isotopic compositions of other planets of Solar system, including Mars and Venus, are markedly different from terrestrial (e.g., deuterium content is ≈5 and ≈100 times higher, respectively), it is far from certain that terrestrial life will thrive in these isotopic conditions. Here we found that Martian deuterium content negatively affected survival of shrimp in semi-closed biosphere on a year-long time scale. Moreover, the bacterium *Escherichia coli* grows slower at Martian isotopic compositions and even slower at Venus’s compositions. Thus, the biological impact of varying stable isotope compositions needs to be taken into account when planning interplanetary missions.

## Introduction

It is well known that isotopic composition of reactants affects the rates of chemical and biochemical reactions. Usually it is assumed that heavy stable isotopes are “slower” due to their higher mass, and their enrichment leads to progressively slower reactions. In the biological context, such retardation means lower rate of biological growth. Indeed, multiple experiments have confirmed this prediction. Shortly after the discovery by Urey *et al*. of deuterium in 1932[1], the biological effects of this heavy isotope of hydrogen have been intensively studied, with negative impact on growth and well-being of many organisms becoming quickly apparent. High concentrations of deuterium in water (“heavy water”) were discovered to reduce protein and nucleic acids synthesis, disturb cell division and affect cellular morphology[2]. Large amounts (≥25%) of deuterium were proven toxic to higher organisms[3]. Although some bacteria are able to adapt to grow in almost pure heavy water[4], their growth becomes markedly slower with as small deuterium concentrations as 1%.

There have been fewer studies on the effect of other heavy stable isotopes in biology than deuterium, with statistically significant alterations noticeable only at high enrichments. Researchers managed to grow several generations of mice in the environment highly enriched with (separately) ^13^C[5], ^18^O[6], and ^15^N[7]. The heavy isotopes of C, N and O are often considered “biologically safe”[6], although recently Turck *et al*. have reported systematic behavioral differences in mice grown on ^15^N diet[7]. They also found that the growth of the bacterium *Escherichia coli* (*E*. *coli*) is slowed down in a media highly enriched with ^15^N[8].

Concerning the low enrichment levels, the data exist almost exclusively on deuterium. Early works (1930s) with deuterium enriched from the normal levels of 150 ppm to ≈600 ppm uncovered a plethora of phenomena[9–17], but most of them were later questioned and/or attributed to experimental artifacts. But in 1970s, Lobyshev et al. have revisited these experiments using much stricter approach and confirmed biological effect of low enrichment. The phenomena were discovered both at the level of individual biochemical reactions (e.g., the Na,K-ATPase activity was found to be increased by 50% at 300–400 ppm D[18,19]) as well as whole organisms[20]. Lobyshev group also studied regeneration of hydroid polyps *Obelia geniculata* in D_2_O added to sea water, and found activation of regeneration by small (≤0.1%) deuterium concentrations as well as strong inhibition at high deuterium concentrations[20]. In relative terms, these effects were much greater than the ratio between the deuterium and hydrogen atoms, which contradicted any conventional explanation. Lobyshev et al. realized that the phenomenon must be collective in nature[19], but could not find a convincing rationalization.

In 1990s, Somlyai et al. have reported that 0.06% deuterium in tissue culture activated the growth of L_929_ fibroblast cell lines[21]. In contrast, water with depleted deuterium (20–120 ppm) suppressed fast-growing cells[21–23]. The latter phenomenon gave rise to the idea of using deuterium-depleted water in anticancer therapy[23]. This line of work is still ongoing, gradually gaining recognition of the mainstream medical community.

The biological impact of low enrichment of heavy stable isotopes is currently becoming of high interest in connection with planning manned planetary missions, including establishing colonies on Mars (future lunar colonies will not face this issue due to the close proximity of lunar isotopic compositions to terrestrial ones). Colonization of Mars is the stated goal of governments[24] as well as global non-profit organizations[25]. Among the many differences Mars has with Earth (pressure, temperature, gravity, solar radiation, composition of atmosphere, near absence of water, etc.), Martian atmosphere is significantly enriched with deuterium (≈840 ppm, or ≈5 times compared to Earth) as well as the heavy isotopes of nitrogen (+68%), carbon and oxygen (+5% each)[26–29].

Here we aimed to investigate how this stable isotope enrichment affects growth of terrestrial organisms. In principle, during billions of years, evolution must have optimized the terrestrial organisms to terrestrial isotopic compositions (the latter changed little on the geological scale). Thus any change in isotopic abundances should result in slower growth. However, recent experiments have shown that this is not necessarily the case[30,31].

## Methodology

As a test organism, *E*. *coli* was chosen, for which we have previously established a very accurate method of measuring the growth parameters[31]. Minimal media with only glucose as a source of carbon ideally suits for the modulation of isotopic abundances. Since manipulation with isotopic composition of nutrients in the growth media may inadvertently cause variations in their concentrations, which would affect the growth parameters, and because the enrichments of D and N are most significant on Mars and Venus, isotopic compositions of only hydrogen in water and nitrogen in an inorganic salt (ammonium chloride) were altered in the media mimicking the conditions on various planets.

In order to separate the effects of enriched deuterium and heavy nitrogen, two types of control media were used–Control-All with all terrestrial isotopic compositions and Control-D—where deuterium level was Martian, while other isotopic compositions were terrestrial. We also simulated Venus’s environment by increasing the deuterium concentration in water to 1.8% (i.e., >100-fold), all other isotopic compositions being terrestrial. The growth parameters at these isotopic compositions, including Control-All, were compared with those at normal terrestrial isotopic compositions (Earth). Although Control-All and Earth had identical isotopic compositions, we treated Control-All as if it were an isotopically different sample to make sure that there is no systematic effect of position, etc., on the growth parameters.

The five used isotopic compositions of the minimal media (Control-All and, identical, Earth, Control-D, Mars and Venus) are given in Table 1.

**Table 1:** Isotopic compositions of the minimal media for conditions of Mars, Venus, Control-All (identical to Earth) and Control-D.

Planet	D	^13^C	^15^N	^18^O
Mars	0.084%	terrestrial	0.588%	terrestrial
Venus	1.75%	terrestrial	terrestrial	terrestrial
Control-All (Earth)	terrestrial	terrestrial	terrestrial	terrestrial
Control-D	0.084%	terrestrial	terrestrial	terrestrial

### E. coli growth experiment

M9 agar plate, *E*. *coli* colony and M9 minimal media (normal) and M9 minimal media enriched with 99.16% ^15^N were prepared as in Ref. 31. *E*. *coli* was grown in an agar plate; one isolated colony was picked with a sterile loop into 5 mL M9 minimal media (normal isotopic condition) and incubated at 37°C while shaking at 200 r.p.m for 5–6 h until it reached its early exponential phase with optical density (O.D_._) around 0.2, measured with Colorimeter WPA CO75 (York, UK).

Four stock solutions (Mars, Venus, Control-All, and Control D) were prepared as shown in S1 Table. First, 6026 μL media with 2.55% ^15^N were prepared by mixing 5893 μL (0.37% ^15^N) and 133 μL (99.16% ^15^N). General procedure of sample preparation on the honeycomb plate was similar to that in Ref. 32. A 22 μL aliquot of the incubated *E*. *coli* culture (O.D. ≈ 0.2) was diluted in 50 mL M9 minimal media of normal isotopic composition (0.37% ^15^N) to prepare *E*. *coli* culture for robot-assisted sample dispensing into 100-well plates.

400 μL M9 minimal media without bacteria were introduced into each of the border wells on the plate to serve as blanks. For each inner well on the plate, 40 μL corresponding stock solution were added manually by pipettes and 360 μL media with bacteria were dispensed with using programmed robotic system (Tecan, Genesis RSP 150, Männedorf, Switzerland). The sample configuration on each plate was as shown in S1 Fig.

After sample preparation, the honeycomb well plates were inserted into the Bioscreen C instrument for incubation at constant temperature. The optical density (OD) of *E*. *coli* in each well was continuously monitored and recorded every six minutes (with wide-band filter of 420–580 nm) with agitation of the plates between the measurements.

The OD on a logarithmic scale was plotted against the growth time to give a growth curve of *E*. *coli*, from which, three growth parameters can extracted: the lag time, the maximum growth rate and the maximum density. Data analysis was performed by using a home-written Excel software as described in detail in Ref. 31.

### BYOES experiment

In this experiment, we tested the effect of deuterium content in water on long-term survival of shrimp in a semi-closed environment. For that we used the BYOES (“build your own eco-system”, S2 Fig) kit developed in 1980s from the NASA spin-off product EcoSphere. BYOES 300 are semi-hermetically closed rectangular plastic containers filled with 300 mL of salted water and containing fibrous sticks, plastic/ceramic beads, algae, microbes and two shrimps. The latter are the only higher organism known to survive for years in such a closed environment. The BYOES 300 was assembled according to its manual. For each BYOES unit, 75 mL of stock solution were added into 225 mL normal water (included in the BYOES kit, with marine salts) to obtained the desired D concentration. Stock solutions were prepared by mixing distilled water containing 25 ppm of deuterium and heavy water (99.9% D) in a proportion according to S2 Table. Five deuterium contents in 5 replicates were prepared: 120 ppm (D-depleted), 150 ppm (D-normal), 300 ppm, 600 ppm and 1200 ppm (all three–D-enriched). The 25 BYOES containers were kept on an office desk for 20 months, arranged in a randomized 5x5 matrix. Every 2–3 weeks the containers were investigated, the shrimps counted (when a shrimp dies, it decomposes and gets consumed by microorganisms within days), and the order of the containers was reshuffled.

## Results

**Fig 1:**
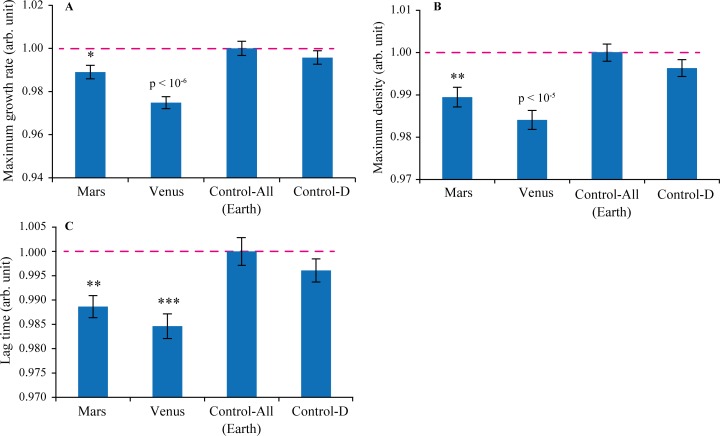
The results of *E*. *coli* growth parameter measurements at 25°C summarizing two independent experiments, each on a separate 100-well plate. (A) Maximum growth rate. (B) Maximum density. (C) Lag time. Usually, more advantageous growth conditions result in faster growth, higher maximum density and shorter lag time.

The results of *E*. *coli* growth at 25°C summarizing two plates with 16 replicate test-control pairs for each condition on each plate are shown in Fig 1. All test results were normalized to the nearby background control. Control-D samples gave lower growth rates and lower maximum density, but without statistical significance. Somewhat surprisingly, the lag phase was shorter (usually, it is a sign of faster growth). This tendency was followed by the Martian and Venus’s compositions, for which the differences were statistically significant (p<0.005 and <0.0005, respectively, in two-tailed, unpaired Student’s t-test). Martian composition also gave significant reduction in both growth rate (p<0.05) and maximum density (p<0.005). As expected, the effect was even stronger for Venus’s composition (p<10^−6^ and <10^−5^, respectively). The relative value of the effect was ≈1% for Mars and ≈2–3% for Venus; such effects are large compared to the precision of measurements (≈0.05% after statistical processing[32]). At 39°C, the temperature of the maximum growth of *E*. *coli*, the differences between the test and control wells were not statistically significant.

### BYOES experiment

**Fig 2:**
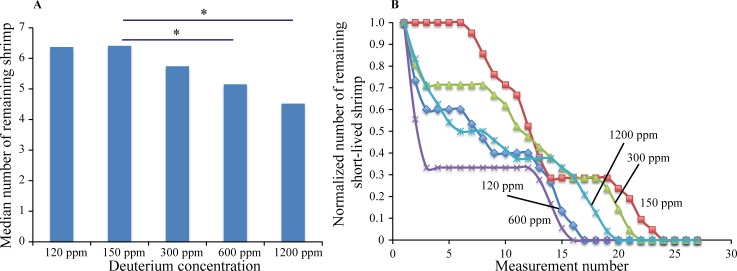
Shrimp survival in BYOES under the period of 20 months. (A) The median number of shrimp in BYOES over the observation period of 20 months as well as the Student’s t-test (two-tailed, unpaired) results for each deuterium content. 150 ppm corresponds to normal terrestrial deuterium content. (B) The survival plots after 3-point smoothing. The number of shrimp that were still alive after 20 months was subtracted for each D content and the obtained value renormalized to day 1.

After 20 months of observations, out of the initial 50 shrimp in all BYOES vessels, only 16 shrimp remained alive (32%). Fig 2A shows the average number of shrimp over the observation period as well as the Student’s t-test (two-tailed, unpaired) results for each deuterium content. Depleted to 120 ppm water as well as enriched to 300 ppm water gave statistically indistinguishable results from normal water, while 600 ppm and especially 1200 ppm water showed statistically significant declines in survival probability (p<0.05 and <0.008, respectively).

The initial age of the shrimp was unspecified, and their individual lifetime is known to vary significantly, from 1–2 years to over a decade. Therefore, a model was additionally considered in which the initial population in each vessel consisted of two groups–a vulnerable group that would die out within the next 20 months, and a long-lived group that would survive much longer regardless of the deuterium content in water. To reduce the influence of the long-lived group on the survival data, the number of shrimp that survived after 20 months was subtracted for each D content, and the data were renormalized. The resultant survival plots (after 3-point smoothing) are shown in Fig 2B. Even in such a model, the normal water content as well as 300 ppm water corresponded to the longest-surviving conditions for the last remaining shrimp, while 600 ppm and 1200 ppm water resulted in faster die-outs (p <0.002 and <0.07, the latter being above the 5% threshold for statistical significance). The reduction of the survival rate by depleted water was borderline significant (p<0.03 before correction for multiple hypotheses), and could be due to statistical fluctuation. If at every time point the remaining number of shrimps are ranked among different deuterium contents, and then the median rank over all time points is taken, 150 and 300 ppm would correspond to ranks 1 and 2, respectively (better overall survival), while 600 ppm and 1200 ppm–to ranks 5 and 3, respectively (poorer survival).

## Discussion

The markedly slower growth of E. coli in Martian conditions is the combined effect of two isotope enrichments–deuterium to 0.084% and ^15^N to 0.59%. Since the proteins contain ≈5.7 times more hydrogen atoms than nitrogen atoms, the molar concentrations of D and ^15^N in Martian samples (assuming equal assimilation degrees) are similar. The biological effect of deuterium at high enrichments is known to be much stronger than that of ^15^N, and thus one could presume that ^15^N enrichment in Martian samples should contribute very little to the overall impact. Indeed, we have observed in our previous work that ≈0.6% ^15^N enrichment performed at normal deuterium conditions makes negligible effect on E. coli growth^37^. At the same time, deuterium enrichment at 0.1% led to a shorter lag time and a somewhat higher maximum density (although both observations were not statistically significant^39^); with the former result being in agreement with the current study.

The data in Fig 1 speak against the above presumption and in favor of the collective effect of the double enrichment. This could be rationalized by recalling the seminal work of the Katz group, who have studied multi-isotope (D together with either ^13^C, ^15^N or ^18^O enrichment, or a combination thereof) substitutions in *Chlorella vulgaris*, and found that all such substitutions result into additional effects in cell size, appearance, growth rate and division compared to deuterium enrichment[33]. Sometimes addition of another heavy isotope to deuterium resulted in amplification of the deuterium effect, and sometimes–in its negation. The authors concluded that “[the] organisms of different isotopic compositions are actually different organisms, to the degree that their isotopic compositions are removed from naturally occurring compositions”. Similarly, here a combined D+^15^N effect turned out to be stronger than either of the individual enrichments.

However, in Katz experiments, the enrichment degree was high (>90%), unlike in our case. Thus, another framework may be needed for rationalization of our results, such as the one provided by the isotopic resonance (IsoRes) phenomenon[32]. The IsoRes hypothesis suggests that the abundances of stable isotopes affect the rates of chemical and biochemical reactions in a nonlinear way, and that at certain isotopic compositions of elements the complexity of the molecules built of these elements decreases. A decreased complexity leads in turn to a change (oftentimes, an increase) in the rate of chemical reactions these molecules engage in. The IsoRes hypothesis is based on a postulate that less complex systems exhibit faster kinetics than equivalent but more complex systems. The system’s complexity is affected by its symmetry (more symmetric systems are simpler), while symmetry (in general meaning) of reactants may be affected by their isotopic composition.

Curiously, average terrestrial isotopic compositions are close to a resonance affecting a large class of amino acids and polypeptides. In IsoRes, the rate of growth is affected by the distance to the nearest strong resonance, which for terrestrial isotopic distributions is the resonance for molecules with Z = 0, where Z = C-(N+H)/2, with C, N and H being the numbers of carbon, nitrogen and hydrogen atoms in the molecule, respectively[34]. The Z = 0 molecules include most amino acid residues, and thus the Z = 0 resonance affects mostly proteins, which are the building blocks for terrestrial life.

Calculations show that at average terrestrial isotopic conditions (150 ppm D and 0.366% ^15^N), the “distance” on the ^15^N scale to the perfect Z = 0-resonance is only 0.009% (i.e., ^15^N abundance needs to be adjusted by only that much, to 0.357%, to achieve the perfect Z = 0-resonance)[35]. This proximity of the terrestrial isotopic compositions to the perfect Z = 0-resonance helps, according to the IsoRes hypothesis, terrestrial life to thrive by slightly increasing the rate of growth for most organisms. When the deuterium content is changed to the Martian 840 ppm D, the perfect Z = 0-resonance moves to 0.422% ^15^N. The terrestrial ^15^N content becomes thus 0.056% away, i.e. six time farther than at the terrestrial D-composition. Since farther resonance means within the IsoRes context lower rate of biological growth, it explains why Control-D samples grew somewhat slower. Large effect was found for Martian samples in which the ^15^N content was 0.588%, i.e. 0.166% away from the Z = 0-resonance. In agreement with the IsoRes hypothesis predictions, the rate of growth further decreased compared to Control-D samples. At the Venus’s deuterium content of 1.8%, the Z = 0 resonance occurs at 2.047% ^15^N, being preceded by other stronger resonances, e.g. at 1.896% of ^15^N. In the vicinity of Venus’s ^15^N content that is similar to Earth’s, there are no strong resonances. Therefore, from the IsoRes point of view, Venus’s conditions are poor for growth of protein-based organisms, in agreement with the observations.

The temperature dependence of the effect magnitude (a significant effect at 25°C and hardly noticeable effect at 39°C) is also in agreement with the IsoRes theory and previous observations[32]. Since the impact of the isotopic resonance is somewhat similar to a temperature increase, the biological growth should accelerate more, in relative terms, when higher temperature leads to faster growth (for *E*. *coli* ≈20–25°C), while at the temperature-growth rate plateau (≈39°C), the relative effect of the isotopic resonance is smaller.

The results of the BYOES experiments can be rationalized through the same framework. Slightly higher deuterium content may not be toxic *per se*, but could instead lead to faster kinetics of most biochemical processes involving proteins (IsoRes theory predicts a strong resonance at ≈300 ppm D[31,32]), which in turn means shorter lifespan. This phenomenon is likely to be responsible for the shorter lifetime at 600 ppm, while at 1200 ppm the general toxicity of deuterium may become the prime reason for the lifespan decline.

## Conclusion

Here we found that the bacterium *Escherichia coli* grow slower at Martian isotopic compositions and even slower at Venus’s compositions. Moreover, Martian deuterium content negatively affects survival of shrimp in semi-closed biosphere on a year-long time scale. Thus, the biological impact of varying stable isotope compositions needs to be taken into account when planning interplanetary missions.

## Supporting Information

S1 Fig Sample configuration on the 100-well honeycomb plate. (PDF)

S2 Fig The assembled BYOES 300. (PDF)

S1 Table Composition of the four stock solutions. (PDF)

S2 Table The composition of the stock solution and the corresponding D content in the BYOES 300 container. (PDF)

S1 File Raw data used for analysis. (RAR)
