# Supplementary figures and images for: On the Effect of Planetary Stable Isotope Compositions on Growth and Survival of Terrestrial Organisms
Source: PLoS One. 2017 Jan 4;12(1):e0169296. doi: 10.1371/journal.pone.0169296 (PMC5215764; doi:10.1371/journal.pone.0169296)

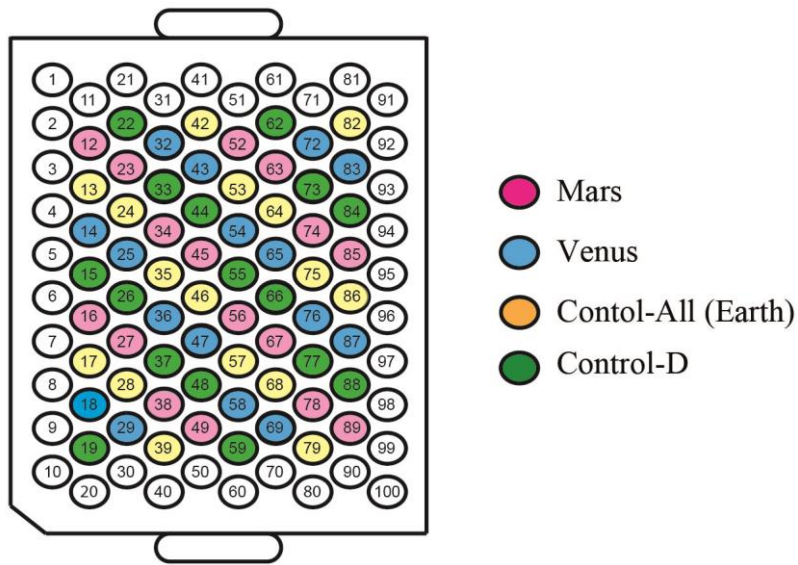

S1 Fig. Sample configuration on the 100-well honeycomb plate.

Supplement: S1 Fig — (PDF) [file pone.0169296.s001.pdf]

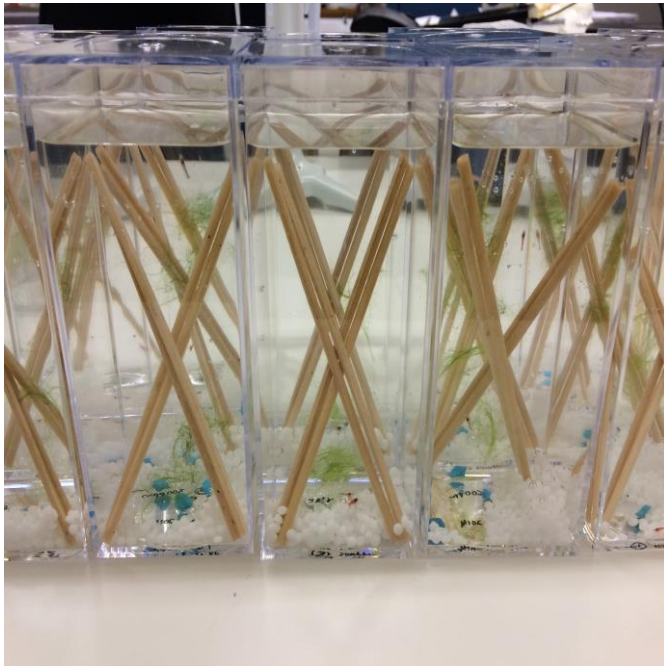

S2 Fig. The assembled BYOES 300.

Supplement: S2 Fig — (PDF) [file pone.0169296.s002.pdf]
