# Supplementary material for: On the Effect of Planetary Stable Isotope Compositions on Growth and Survival of Terrestrial Organisms
Source: PLoS One. 2017 Jan 4;12(1):e0169296. doi: 10.1371/journal.pone.0169296 (PMC5215764; doi:10.1371/journal.pone.0169296)
Supplement: S1 Table — (PDF) [file pone.0169296.s003.pdf]

Supplementary Materials

for

On the Effect of Planetary Stable Isotope Compositions on Growth and Survival of Terrestrial Organisms

*Xueshu Xie and Roman A. Zubarev**

Division of Physiological Chemistry I, Department of Medical Biochemistry and Biophysics, Karolinska Institutet, SE-17 177 Stockholm, Sweden

*Corresponding author: Roman.Zubarev@ki.se phone/fax +46 8 524 87 594

S1 Table. Composition of the four stock solutions.

Stock solution	M9 media, µL	Heavy water (99.16% D), µL	Milli-Q water, µL
Mars	4831.7 (2.55% ^{15}N media)	975.3	40
Venus	4831.7 (normal)	1015.3	0
Control-All (Earth)	4831.7 (normal)	0	1015.3
Control-D	4831.7 (normal)	40	975.3
