# Supplementary material for: On the Effect of Planetary Stable Isotope Compositions on Growth and Survival of Terrestrial Organisms
Source: PLoS One. 2017 Jan 4;12(1):e0169296. doi: 10.1371/journal.pone.0169296 (PMC5215764; doi:10.1371/journal.pone.0169296)
Supplement: S2 Table — (PDF) [file pone.0169296.s004.pdf]

S2 Table. The composition of the stock solution and the corresponding D content in the BYOES 300 container.

**D composition in stock solution (%)**	**Total volume, µL**	**25 ppm water, µL**	**Heavy water, 99.9% D, µL**	**D content in the BYOES container, ppm**
0.003	380,000	379981.0	19.0 (10% D)	120
0.015	380,000	379952.5	47.5	150
0.075	380,000	379724.2	275.8	300
0.195	380,000	379267.7	732.3	600
0.435	380,000	378354.8	1645.2	1200
